# Tracking Response Dynamics of Sequential Working Memory in Patients With Mild Parkinson’s Disease

**DOI:** 10.3389/fpsyg.2021.631672

**Published:** 2021-02-19

**Authors:** Guanyu Zhang, Jinghong Ma, Piu Chan, Zheng Ye

**Affiliations:** ^1^Institute of Psychology, Chinese Academy of Sciences, Beijing, China; ^2^Department of Psychology, University of Chinese Academy of Sciences, Beijing, China; ^3^Department of Neurology, Xuanwu Hospital of Capital Medical University, Beijing, China; ^4^Department of Neurology and Neurobiology, National Clinical Research Center for Geriatric Disorders, Xuanwu Hospital of Capital Medical University, Beijing, China; ^5^Institute of Neuroscience, Key Laboratory of Primate Neurobiology, Center for Excellence in Brain Science and Intelligence Technology, Chinese Academy of Sciences, Shanghai, China; ^6^Shanghai Center for Brain Science and Brain-Inspired Intelligence Technology, Shanghai, China

**Keywords:** Parkinson’s disease, dopamine, sequential working memory, mouse tracking, dopamine D2 receptor agonist

## Abstract

The ability to sequence thoughts and actions is impaired in Parkinson’s disease (PD). In PD, a distinct error pattern has been found in the offline performance of sequential working memory. This study examined how PD’s performance of sequential working memory unfolds over time using mouse tracking techniques. Non-demented patients with mild PD (*N* = 40) and healthy controls (*N* = 40) completed a computerized digit ordering task with a computer mouse. We measured response dynamics in terms of the initiation time, ordering time, movement time, and area under the movement trajectory curve. This approach allowed us to distinguish between the cognitive processes related to sequence processing before the actual movement (initiation time and ordering time) and the execution processes of the actual movement (movement time and area under the curve). PD patients showed longer initiation times, longer movement times, and more constrained movement trajectories than healthy controls. The initiation time and ordering time negatively correlated with the daily exposure to levodopa and D2/3 receptor agonists, respectively. The movement time positively correlated with the severity of motor symptoms. We demonstrated an altered temporal profile of sequential working memory in PD. Stimulating D1 and D2/3 receptors might speed up the maintenance and manipulation of sequences, respectively.

## Introduction

The ability to sequence thoughts and actions is impaired even in the early stages of Parkinson’s disease (PD). PD patients with mild to moderate clinical symptoms tend to misunderstand the temporal relation of events expressed out of chronological order, no matter whether they have been treated with dopaminergic drugs (Natsopoulos et al., 1991; Al-Khaled et al., 2012). They have difficulty organizing verbal sequences (Bublak et al., 2002; Lewis et al., 2005) and planning sequential moves (Morris et al., 1988; Owen et al., 1992). Recently, we analyzed the offline performance of sequential working memory and found a distinct error pattern in PD (Ma et al., 2019). PD patients tended to recall more items too early than healthy controls when rearranging sequential items (i.e., more anticipation errors). In this study, we took a different angle: examing how PD’s performance of sequential working memory unfolds in real-time with mouse tracking techniques.

The mouse tracking technique is built upon the idea that the dynamics of the hand can reflect the dynamics of the mind (Spivey et al., 2005; Freeman and Ambady, 2010). Measuring the trajectories of how participants move a computer mouse into one of the multiple response alternatives at a sampling rate of 40–120 Hz could potentially reveal the real-time evolution of internal cognitive processes (for a review, see Freeman et al., 2011). This technique has been used to understand various cognitive processes in healthy adults and clinical populations, from language (e.g., spoken word and sentence comprehension, see Spivey et al., 2005; Dale and Duran, 2011), attention and motor control (Xiao and Yamauchi, 2017; Benedetti et al., 2020), to social cognition (e.g., social and race categorization, see Dale et al., 2007; Freeman et al., 2008).

The mouse tracking technique allows researchers to separate the cognitive processes and cognitive aspects of motor programming before the actual movement (measured as the initiation time) and the execution processes of the actual movement [measured as the movement time and the area under the movement trajectory curve (AUC)]. Therefore, it has the potential to dissociate PD’s deficits in the cognitive versus motor aspects of sequence learning and memory. For example, in the study of Ruitenberg et al. (2016) PD patients learned a sequence of mouse movements through trial and error in multiple sessions with and without dopaminergic drugs. With mouse tracking, Ruitenberg et al. (2016) found that the motor aspect of sequence learning was improved (larger learning effects in the movement trajectory), whereas the cognitive aspect was impaired by dopaminergic drugs (smaller learning effects in the initiation time). Splitting the cognitive and motor aspects might increase the chance to find significant associations between task performance and clinical features. For example, Leontyev and Yamauchi (2019) found that compared to a traditional button-pressing version of the stop-signal task, the mouse tracking version revealed a stronger association between movement measures and impulsivity in adults with attention-deficit/hyperactivity disorder (ADHD) and increased the prediction accuracy of machine learning models.

**FIGURE 1:**
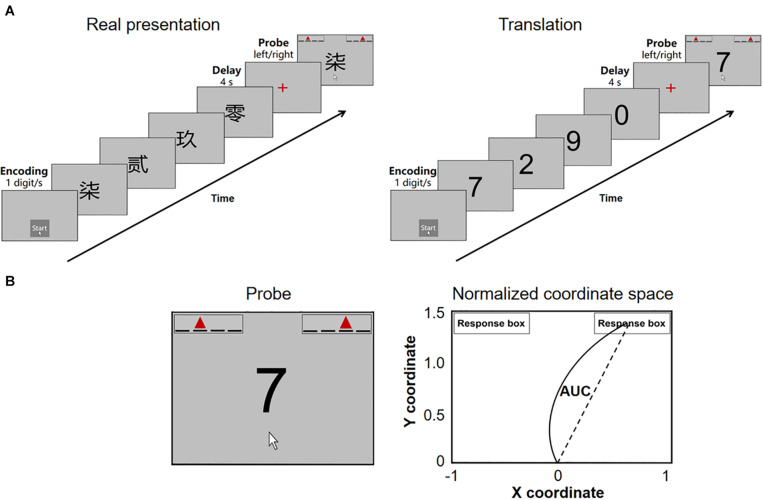
**(A)** The digit ordering task with mouse tracking. **(B)** The probe and standard coordinate space for parameter estimation. At the probe stage, participants see a digit probe and two response boxes. Each response box contains four underlines and a red triangle. The underlines indicate our ordinal positions from left to right. The red triangle indicates the position of the digit probe. AUC, the area under the curve.

This study investigated the temporal profile of sequential working memory performance in non-demented patients with mild PD by combining mouse tracking with a computerized digit ordering task (Figure 1A). In this task, participants had either to reorder randomized digits in ascending order (“reorder and recall” trials) or simply to recall them in the original order (“pure recall” trials). They then moved the computer mouse to select one of the two response alternatives. First, we distinguished between the task’s cognitive and motor aspects by measuring the initiation time, movement time, and AUC. In “pure recall” trials, the initiation time reflected at least three cognitive stages: (1) maintenance of sequences, (2) selection of a response, and (3) cognitive aspects of motor programming. Second, we isolated the manipulation of sequences by calculating the initiation time difference between “reorder and recall” and “pure recall” trials (ordering time). Third, we explored the potential role of dopamine in the maintenance versus manipulation of sequences, asking whether the initiation time and/or the ordering time correlate with the daily exposure to levodopa and/or D2/3 receptor agonists.

## Materials and Methods

This study was approved by the ethics committee of the Xuanwu Hospital following the Declaration of Helsinki. Each participant signed a written informed consent before participating in this study.

### Patients and Clinical Assessments

We included 40 patients with idiopathic PD [Movement Disorder Society Clinical Diagnostic Criteria for PD (Postuma et al., 2015)] at the Xuanwu Hospital Research and Clinical Center for PD between 2018–2019. Inclusion criteria were (1) Hoehn and Yahr Stages 1–2; (2) age 40–75 years; (3) education more than 8 years; (4) Mandarin Chinese speaking; (5) right-handed; (6) using the computer mouse in daily life. Exclusion criteria were (1) a history of other neurological diseases (e.g., epilepsy, stroke, or brain injury); (2) alcohol or drug abuse; (3) possible current depression (Beck Depression Inventory-II, BDI-II > 7) or taking anti-depressants; (4) possible dementia (Montreal Cognitive Assessment, MoCA < 21/30) or taking anti-dementia drugs; (5) working memory spans lower than four (digit span forward test and adaptive digit ordering test). Eight additional patients were excluded because they had a MoCA < 21/30 (*N* = 5) or a working memory span lower than four (*N* = 3).

Table 1 shows demographic, clinical, and neuropsychological data of the patients and healthy controls. All patients were assessed on their regular anti-parkinsonian drugs, including levodopa (*N* = 20), pramipexole (*N* = 14), selegiline (*N* = 10), piribedil (*N* = 6), amantadine (*N* = 4), entacapone (*N* = 2), and rasagiline (*N* = 1). The levodopa equivalent dose was calculated using the equation of Tomlinson et al. (2010). The actual levodopa dose and the levodopa equivalent dose for D2/3 receptor agonists were calculated separately. The severity of motor and non-motor symptoms was evaluated with the Movement Disorder Society-sponsored revision of the Unified Parkinson’s Disease Rating Scale (MDS-UPDRS) III (Motor Examination) and I subscales (Non-motor Experiences of Daily Living). The severity of sleep dysfunction was evaluated with the REM Sleep Behavior Disorder Screening Questionnaire and Epworth Sleep Scale.

**TABLE 1:** Demographic, clinical, and neuropsychological data of the patients and healthy controls (means, standard deviations, and group differences).

**Features/Measures**	**Patients (*N* = 40)**	**Healthy controls (*N* = 40)**	**Group differences (*p-*values)**
Female: Male	20:20	20:20	1.000
Age (years)	57.5 (7.5)	56.9 (7.4)	0.731
Education (years)	12.2 (2.7)	12.8 (1.9)	0.229
**Motor symptoms**			
Hoehn and Yahr Stage	1.4 (0.5)		
MDS-UPDRS III: Motor examination	17.9 (9.1)		
Disease duration (years)	1.9 (2.8)		
Duration of motor symptoms (years)	3.0 (2.7)		
**Cognition**			
Montreal cognitive assessment	25.8 (2.0)	26.9 (1.0)	0.005*
Digit span forward test	8.0 (0.9)	7.9 (0.8)	0.597
Adaptive digit ordering test	5.1 (1.0)	5.6 (1.1)	0.028
**Other non-motor functions**			
MDS-UPDRS I: Non-motor experiences of daily living	5.2 (3.8)		
Beck depression inventory-II	3.6 (2.4)	2.4 (2.1)	0.018
REM Sleep behavior disorder screening questionnaire	4.7 (2.9)	2.7 (2.0)	0.001*
Epworth sleep scale	4.4 (4.3)	6.0 (3.6)	0.074
**Levodopa equivalent dose**			
Total (mg/day)	279.1 (268.4)		
Levodopa (mg/day)	186.6 (223.9)		
D2/3 receptor agonists (mg/day)	53.1 (61.1)		

MDS-UPDRS, the Movement Disorder Society-sponsored revision of the Unified Parkinson’s Disease Rating Scale; Group differences, *p* values of two-sample *t*-tests, or Chi-square test as appropriate; asterisks (*), a significant difference (two-tailed, *p* < 0.006 Bonferroni correction for nine tests).

### Healthy Control Subjects

We included 40 age-, education-, and handedness-matched healthy controls from local communities. Exclusion criteria were (1) a history of significant neurological or psychiatric disorders; (2) alcohol or drug abuse; (3) possible current depression; (4) possible dementia or mild cognitive impairment (MoCA < 26/30); (5) working memory spans lower than four. They completed the same assessments for cognition, mood, and sleep as the patients.

### Experimental Design and Procedure

We optimized the digit ordering task (Ye et al., 2020, 2021) for mouse tracking (Figure 1A). The task was programmed with the Mouse Tracker software (Freeman and Ambady, 2010) running in the Windows 10 system. Participants performed the task using a Logitech M100R mouse, with the cursor speed set at the 6/11 default mode and a mouse/cursor mapping of 1:1.

The task included interleaved “pure recall” (60 trials) and “reorder and recall” trials (64 trials). Participants triggered each trial by clicking the mouse over a start button (1.5 cm × 1.5 cm). They were presented with a sequence of four different digits written in Traditional Chinese (4 cm × 4 cm) and asked to remember the digits in ascending order through a short delay. In “pure recall” trials, the digits were presented in ascending order (e.g., *0-2-7-9*). In “reorder and recall” trials, the digits were fully randomized, and participants always had to reorder them (e.g., *7-2-9-0*).

After the delay, participants saw a digit probe and two response boxes (1.5 cm × 6 cm, Figure 1B). The target box indicated the target position of the digit probe (e.g., for probe *7* in sequence *7-2-9-0*, the target position is *third*). The distractor box indicated an incorrect position preceding the target position (anticipation errors, e.g., the position *second*). It is found that anticipation errors are the most frequent error types in digit ordering tests (Ma et al., 2019). Participants were asked first to choose the correct response box in mind and then to move the mouse onto the response box and click the mouse to register their choice with the right hand as quickly as possible. There was no response time limit. The mouse cursor was automatically relocated to the start button when the probe occurred. The distance between the start button and the center of either response box was 17 cm. During the movement, the mouse cursor location (*x*, *y*) was recorded at a sampling rate of 70 Hz.

Participants completed a practice block (4 min) and four experimental blocks (8 min each). The trial sequence of each experimental block was designed to ensure that (1) the transition probability of the trial type was evenly balanced; (2) the digits were not repeated in any two consecutive trials; (3) the location of the correct response box was repeated no more than three times in consecutive trials.

We first measured the percentage of correct responses (accuracy). We then normalized mouse trajectories from the real space to the standard coordinate space (Figure 1B) and flipped the trajectories toward the left side to the right side. The standard coordinate space ranges between [−1, 1] on the *x*-axis and [0, 1.5] on the *y*-axis (Freeman and Ambady, 2010). We measured the initiation time, ordering time, movement time, and AUC. The initiation time was the interval between the onset of the probe and that of the mouse movement. In “pure recall” trials, at least three cognitive stages were involved in this interval: (1) maintenance of sequences, (2) selection of a response, and (3) cognitive aspects of motor programming. In “reorder and recall” trials, the manipulation of sequences (ordering) was additionally involved. We calculated the ordering time as the initiation time difference between “reorder and recall” and “pure recall” trials. The movement time was the interval between the onset of the mouse movement and the mouse click. The AUC was the geometric area between the actual trajectory and the idealized straight trajectory, as the percentage of total standard coordinate space. The movement time and AUC reflected the execution processes of the actual movement.

### Statistical Analysis

Data were analyzed with IBM SPSS Statistics 20. First, we examined whether PD patients responded less accurately or more slowly (longer initiation time or movement time) or through a more constrained mouse trajectory (smaller AUC) than healthy controls using repeated-measures ANOVAs (one-tailed). The ANOVA had two factors, Trial Type (“pure recall”, “reorder and recall”) and Group (PD, healthy control). We also examined whether PD patients manipulated sequences more slowly (longer ordering time) than healthy controls using a two-sample *t*-test (one-tailed). Significance was considered at *p* < 0.01 (Bonferroni correction for four ANOVAs and one *t*-test).

Second, we examined whether the cognitive aspect (the initiation time and ordering time) were related to the motor aspect (the movement time and AUC). In particular, we correlated the initiation time of “pure recall” trials and the ordering time (“reorder and recall” versus “pure recall” trials) with the movement time of “pure recall” trials. As a validity check, we also correlated the movement time of “pure recall” trials with the AUC of “pure recall” trials. Significance was considered at *p* < 0.017 (Bonferroni correction for three correlation tests).

Third, we examined whether the cognitive aspect (the initiation time and ordering time) was related to dopaminergic stimulation. We correlated the initiation time of “pure recall” trials and the ordering time with the actual levodopa dose and levodopa equivalent dose of D2/3 receptor agonists. As a validity check, we also correlated the movement time of “pure recall” trials with the severity of motor symptoms (MDS-UPDRS III score). Significance was considered at *p* < 0.017 (Bonferroni correction for three correlation tests).

### Data Availability

Data have been uploaded to the figshare database https://figshare.com/articles/dataset/XW_Data_2018-2019_xls/13642874.

## Results

### Group Differences in Response Dynamics

Both PD patients and healthy controls performed the digit ordering task attentively. For “pure recall/reorder and recall” trials, the accuracy was 98.7/97.3% in PD patients and 98.7/97.1% in healthy controls. The main effect of Trial Type was found [*F*(1,78) = 17.69, *p* < 0.001, η*_*p*_*^2^ = 0.19] but no main effect of Group or interaction between Group and Trial Type (*F*s < 1). The absence of a group difference in accuracy is not unexpected. This task was designed to maximize correct trials in both groups using a relatively low memory load.

**FIGURE 2:**
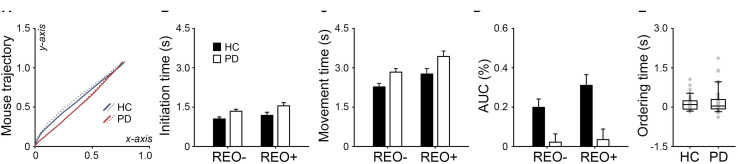
**(A)** Normalized mouse trajectories for “pure recall” (REO-, solid line) and “reorder and recall” trials (REO+, dotted line) in PD patients and healthy controls (HC). The trajectories toward the left side were flipped to the right side. PD patients had **(B)** longer initiation times, **(C)** longer movement times, and **(D)** smaller area under the trajectory curve (AUC) than healthy controls. Error bars indicate standard errors. **(E)** PD patients did not show longer ordering times than healthy controls. Gray dots indicate individual participants.

Figure 2A shows normalized mouse trajectories of each group in the standard coordinate space. For the initiation time (Figure 2B), the main effects of Group [*F*(1,78) = 5.83, *p* = 0.009, η*_*p*_*^2^ = 0.07] and Trial Type were found [*F*(1,78) = 15.78, *p* < 0.001, η*_*p*_*^2^ = 0.17], but no interaction (*F* < 1). There was a similar pattern for the movement time (Figure 2C): significant main effects of Group [*F*(1,78) = 7.22, *p* = 0.005, η*_*p*_*^2^ = 0.09] and Trial Type [*F*(1,78) = 44.19, *p* < 0.001, η*_*p*_*^2^ = 0.36], but no interaction (*F* < 1). For the AUC (Figure 2D), the interaction between Group and Trial Type was found [*F*(1, 78) = 9.46, *p* = 0.002, η*_*p*_*^2^ = 0.11], in addition to the main effects of Group [*F*(1,78) = 11.80, *p* < 0.001, η*_*p*_*^2^ = 0.13] and Trial Type [*F*(1,78) = 15.33, *p* < 0.001, η*_*p*_*^2^ = 0.16]. However, there was no group difference in the ordering time (*t* < 1, Figure 2E). The results suggest that participants were generally slower in “reorder and recall” than “pure recall” trials. PD patients were slower than healthy controls in both cognitive and motor aspects. Healthy controls tended to deviate more from the optimal trajectory in “reorder and recall” than “pure recall” trials. However, PD patients were largely constrained in the mouse trajectory regardless of the trial type.

### No Correlation Between Cognitive and Motor Aspects

**FIGURE 3:**
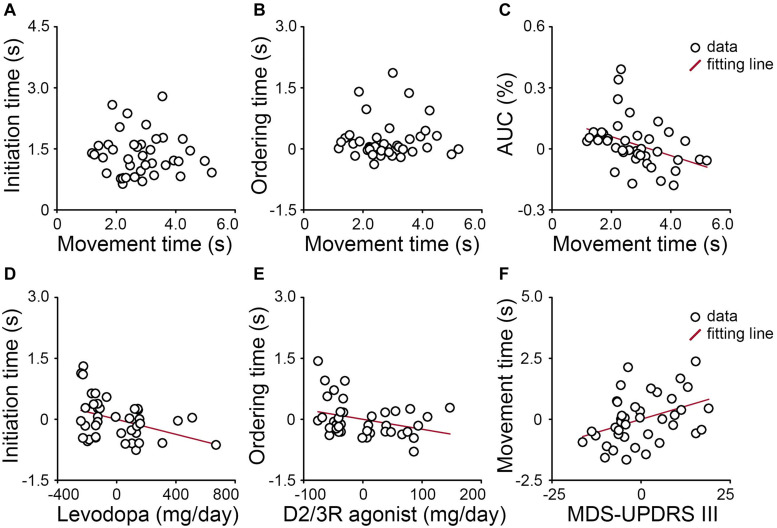
In PD patients, neither **(A)** the initiation time of “pure recall” trials nor **(B)** the ordering time (“reorder and recall” versus “pure recall” trials) was correlated with the movement time of “pure recall” trials. **(C)** For “pure recall” trials, the area under the trajectory curve (AUC) was negatively correlated with the movement time. **(D)** The initiation time of “pure recall” trials was negatively correlated with the actual levodopa dose. **(E)** The ordering time was negatively correlated with the levodopa equivalent dose for D2/3 receptor agonists. **(F)** The movement time of “pure recall” trials was positively correlated with the severity of motor symptoms (MDS-UPDRS III score). Values are demeaned.

Figures 3A,B shows no correlation between cognitive and motor aspects in PD patients. Neither the initiation time of “pure recall” trials (*r* = −0.05, *p* = 0.780, Figure 3A) nor the ordering time (“reorder and recall” versus “pure recall” trials) was correlated with the movement time of “pure recall” trials (*r* = 0.04, *p* = 0.807, Figure 3B).

For “pure recall” trials, the movement time was correlated with the AUC (*r* = −0.39, *p* = 0.013, Figure 3C). PD patients who moved the mouse more slowly tended to move through a more constrained trajectory.

### Relationships Between Clinical Features and Cognitive and Motor Aspects

Figures 3D–F shows relationships between the cognitive aspect and dopamine and between the motor aspect and disease severity. The initiation time of “pure recall” trials was negatively correlated with the actual levodopa dose (*r* = −0.40, *p* = 0.014) when the effects of the levodopa equivalent dose for D2/3 receptor agonists and the severity of non-motor symptoms (MDS-UPDRS I score) were controlled (Figure 3D). By contrast, the ordering time (“reorder and recall” versus “pure recall” trials) was marginally negatively correlated with the levodopa equivalent dose for D2/3 receptor agonists (*r* = −0.33, *p* = 0.045) when the effects of the actual levodopa dose and the severity of non-motor symptoms were controlled (Figure 3E). The movement time of “pure recall” trials was positively correlated with the severity of motor symptoms (*r* = −0.40, *p* = 0.013) when the effect of the total levodopa equivalent dose was controlled (Figure 3F). In other words, PD patients who received higher daily doses of levodopa or D2/3 receptor agonists tended to be faster cognitively. PD patients who had more severe motor symptoms tended to move more slowly. We explored correlations between mouse tracking parameters and other clinical features and presented as Supplementary Materials.

## Discussion

This study demonstrated an altered temporal profile of sequential working memory performance in non-demented patients with mild PD using mouse tracking. In general, both PD patients and healthy controls performed the digit ordering task with lower accuracy, longer initiation times, longer movement times, and more deviated movement trajectories when rearranging sequential digits (“reorder and recall” versus “pure recall” trials). This observation suggests that manipulating sequences is cognitively demanding and has a spill-over effect on the motor aspect. It is consistent with our neuroimaging findings that the lateral prefrontal and posterior parietal regions were more activated and more strongly connected with the supplementary motor area for “reorder and recall” than “pure recall” trials (Ye et al., 2020).

Our primary finding is that PD patients were impaired in the cognitive and motor aspects of sequential working memory performance. PD patients exhibited longer initiation times, longer movement times, and more constrained movement trajectories than healthy controls. In PD, the cognitive aspect (the initiation time and ordering time) was related to the daily exposure to dopaminergic drugs (levodopa and D2/3 receptor agonists) but not to the motor aspect (the movement time). It implies a potential role of dopamine in sequential working memory.

The cognitive aspect is often measured indirectly through a cognitive subtraction method or memory load manipulation. The subtraction method assumes that a single cognitive process can be inserted into a preexisting set of cognitive processes without affecting them. For example, Zimmermann et al. (1992) asked early and advanced PD patients to perform a simple reaction task and a choice reaction time task and calculated the decision time by subtracting the simple reaction time from the choice reaction time. Both early and advanced PD patients showed longer decision times than healthy controls. Using a similar design, Jokinen et al. (2013) found that PD’s slowed calculation was related to the striatal dopaminergic dysfunction. We also employed the subtraction concept to isolate the manipulation of sequences.

An alternative experimental strategy is to manipulate the number or presentation speed of items to be remembered (Press et al., 2002; Sawamoto et al., 2002; Hanakawa et al., 2017). For example, Sawamoto et al. (2002) asked PD patients to mentally move a marker on a checkerboard following a series of instructions. The instructions were displayed sequentially at a speed of 0.4–1.8 Hz. PD patients became less accurate than healthy controls when the presentation speed reached or exceeded 1 Hz. Using a similar design, Hanakawa et al. (2017) showed that PD’s slowed calculation, imagery, and movement were related to the dysfunction of the language, premotor, and motor loops of the cortico-basal ganglia-thalamo-cortical circuits, respectively.

Dopamine is known for its modulatory role in visuospatial working memory, with D1 receptors mediating the maintenance of item information (e.g., color, location) and D2 receptors mediating the manipulation of task-relevant information (e.g., updating). However, neural representations of sequential information might differ from those of item information. Previous electrophysiological studies showed that prefrontal theta oscillations increased for maintaining sequences, whereas prefrontal gamma and posterior alpha oscillations increased for maintaining item information (Hsieh et al., 2011; Roberts et al., 2013). Moreover, neuroimaging studies showed that the lateral prefrontal cortex and intraparietal sulcus were more activated for maintaining sequential information than item information (Marshuetz et al., 2000; Attout et al., 2019). Therefore, it is unclear whether dopamine plays a similar role in sequential working memory. Our work showed that stimulating D2/3 receptors might reduce PD’ tendency to make transposition errors (especially anticipation errors) (Ma et al., 2019) and speed up ordering processes. We propose that D2/3 receptors are involved in manipulating sequences, even though there are conflict findings from human psychopharmacological studies (Cooper et al., 1992; Dodds et al., 2009; Fallon et al., 2017). Future research is needed to test this hypothesis with pharmacological neuroimaging.

This study has limitations. The mouse tracking technique does not separate pure cognitive processes (e.g., working memory) from cognitive aspects of motor programming. It is unclear whether PD’s longer initiation time reflected slowing in encoding, storing, and retrieving sequences, motor planning, or both.

In conclusion, we demonstrated PD’s deficits in sequential working memory from a temporal perspective. PD patients exhibited longer initiation times, longer movement times, and more constrained movement trajectories than healthy controls. It suggests that the disease effect might occur in both cognitive and motor aspects of sequential working memory. It would be interesting to investigate whether the disease effect on the cognitive aspect might exist in the prodromal stage or could serve as a biomarker for predicting PD conversion. Moreover, in PD, the initiation time and ordering time were negatively correlated with the daily exposure to levodopa and D2/3 receptor agonists, respectively. It implies that D1 and D2 receptors might play different roles in maintaining and manipulating sequences.

## Data Availability Statement

The datasets presented in this study can be found in online repositories. The names of the repository/repositories and accession number(s) can be found below: https://figshare.com/articles/dataset/XW_Data_2018-2019_xls/13642874.

## Ethics Statement

The studies involving human participants were reviewed and approved by the ethics committee of the Xuanwu Hospital. The patients/participants provided their written informed consent to participate in this study.

## Author Contributions

GZ and ZY designed the study and analyzed the data and wrote the original draft of the manuscript. GZ, JM, and PC collected the data. JM and PC reviewed and edited the manuscript. All authors approved the submitted version.

## Conflict of Interest

The authors declare that the research was conducted in the absence of any commercial or financial relationships that could be construed as a potential conflict of interest.
