# Supplementary material for: Tracking Response Dynamics of Sequential Working Memory in Patients With Mild Parkinson’s Disease
Source: Front Psychol. 2021 Feb 19;12:631672. doi: 10.3389/fpsyg.2021.631672 (PMC7933003; doi:10.3389/fpsyg.2021.631672)
Supplement: Supplementary file 1 [file Data_Sheet_1.pdf]

Supplementary information for

Tracking response dynamics of sequential working memory in patients with mild Parkinson's disease

Guanyu Zhang [1,2], Jinghong Ma [3], Piu Chan [4], Zheng Ye [5,6,*]

[1]*Institute of Psychology, Chinese Academy of Sciences, Beijing, China*

[2]*Dept. of Psychology, University of Chinese Academy of Sciences, Beijing, China*

[3]*Dept. of Neurology, Xuanwu Hospital of Capital Medical University, Beijing, China*

[4]*Dept. of Neurology and Neurobiology, National Clinical Research Center for Geriatric Disorders, Xuanwu Hospital of Capital Medical University, Beijing, China*

[5]*Institute of Neuroscience, Key Laboratory of Primate Neurobiology, Center for Excellence in Brain Science and Intelligence Technology, Chinese Academy of Sciences, Shanghai, China*

[6]*Shanghai Center for Brain Science and Brain-Inspired Intelligence Technology, Shanghai 201210, China*

G.Z. and J.M. equally contributed to this work.

*Corresponding authors:

Zheng Ye, Institute of Neuroscience, Center for Excellence in Brain Science and Intelligence Technology, Chinese Academy of Sciences, Yueyang Road 320, Shanghai 200031, China, E-mail: yez@ion.ac.cn

1. Relationships between response dynamics and clinical features

We explored correlations between response dynamics and other clinical features (e.g., disease duration, duration of motor symptoms). Significance was considered at $p<0.002$ (Bonferroni correction for twenty-four tests).

Table S1 presents the correlation coefficients between mouse tracking parameters and clinical features. There was no significant correlation.

Table S1: Correlations between mouse tracking parameters and clinical features (r and p values)

Mouse tracking parameters		Disease duration	Duration of motor symptoms	MDS-UPDRS Part I	BDI	RBDSQ	ESS
Pure recall	IT	r=-0.21<br>p=0.19	r=-0.25<br>p=0.12	r=0.12<br>p=0.46	r=-0.19<br>p=0.24	r=-0.16<br>p=0.33	r=-0.17<br>p=0.28
	MT	r=0.13<br>p=0.44	r=0.15<br>p=0.34	r=0.06<br>p=0.73	r=0.21<br>p=0.20	r=0.14<br>p=0.40	r=0.30<br>p=0.06
	AUC	r=0.00<br>p=0.99	r=-0.06<br>p=0.72	r=0.13<br>p=0.43	r=0.00<br>p=0.99	r=-0.13<br>p=0.41	r=-0.05<br>p=0.77
Ordering time		r=-0.13<br>p=0.43	r=-0.16<br>p=0.31	r=0.27<br>p=0.10	r=0.03<br>p=0.85	r=0.06<br>p=0.73	r=-0.10<br>p=0.54

Note: IT, initiation time; MT, movement time; AUC, area under the curve; MDS-UPDRS: Movement Disorder Society-sponsored revision of the Unified Parkinson's Disease Rating Scale; BDI, Beck Depression Inventory-II; RBDSQ, REM Sleep Behaviour Disorder Screening Questionnaire; ESS, Epworth Sleep Scale.

2. Distribution analysis of the initiation and movement times

We performed an exploratory distribution analysis divided by quartiles. First, we divided PD patients into three subgroups (fastest, medium, slowest) according to the initiation time averaged across trial types. For each subgroup, we examined whether the patients had longer initiation times than healthy controls using repeated-measures ANOVAs (one-tailed, p<0.017 Bonferroni correction for three ANOVAs). The ANOVA had two factors, Group (PD subgroup, healthy control) and Trial Type ('pure recall', 'reorder & recall').

Fig.S1A shows the distribution analysis of the initiation time. For the slowest PD subgroup *versus* healthy controls, the main effects of Group (F(1, 48)=39.47, p<0.001, η_p^2=0.45) and Trial Type (F(1, 48)=35.00, p<0.001, η_p^2=0.42) and the interaction between Group and Trial Type were found (F(1, 48)=15.88, p<0.001, η_p^2=0.25). For the medium PD subgroup *versus* healthy controls, the main effect of Trial Type was found (F(1, 58)=10.85, p=0.001, η_p^2=0.16), but no main effect of Group (F(1, 58)=3.14, p=0.041, η_p^2=0.05) or interaction (F<1). For the fastest PD subgroup *versus* healthy controls, there was no main effect of Group (F(1, 48)=4.47, p=0.02, η_p^2=0.09) or Trial Type (F(1, 48)=1.02, p=0.16, η_p^2=0.02) or interaction (F(1, 48)=4.25, p=0.02, η_p^2=0.08). The slowest PD subgroup had longer initiation times than health controls, especially for 'reorder & recall' trails.

Second, we divided PD patients into three subgroups (fastest, medium, slowest) according to the movement time averaged across trial types. For each subgroup, we examined whether the patients had longer movement times than healthy controls using repeated-measures ANOVAs (one-tailed, p<0.017 Bonferroni correction for three ANOVAs). The ANOVA had two factors, Group (PD subgroup, healthy control)

and Trial Type ('pure recall', 'reorder & recall').

Fig.S1B shows the distribution analysis of the movement time. For the slowest PD subgroup *versus* healthy controls, the main effects of Group ($F(1, 48)=69.91$, $p<0.001$, $\eta_p^2=0.59$) and Trial Type ($F(1, 48)=41.96$, $p<0.001$, $\eta_p^2=0.47$) and the interaction between Group and Trial Type were found ($F(1, 48)=9.18$, $p=0.002$, $\eta_p^2=0.16$). For the medium PD subgroup *versus* healthy controls, the main effects of Group ($F(1, 58)=5.48$, $p=0.012$, $\eta_p^2=0.09$) and Trial Type were found ($F(1, 58)=52.26$, $p<0.001$, $\eta_p^2=0.47$), but not interaction ($F<1$). For the fastest PD subgroup *versus* healthy controls, the main effects of Group ($F(1, 48)=8.08$, $p=0.004$, $\eta_p^2=0.14$) and Trial Type were found ($F(1, 48)=13.66$, $p<0.001$, $\eta_p^2=0.22$), but not interaction ($F(1, 48)=4.11$, $p=0.024$, $\eta_p^2=0.08$). The slowest PD subgroup had longer movement times than healthy controls, especially for 'reorder & recall' trials. The medium PD subgroup had longer movement times regardless of the trial type.

Fig.S1: The distribution analysis of (A) the initiation time and (B) movement time in PD patients and healthy controls (HC). The patients were divided into three subgroups according to the initiation or movement time. Error bars indicate standard errors. REO-, pure recall; REO+, reorder & recall.

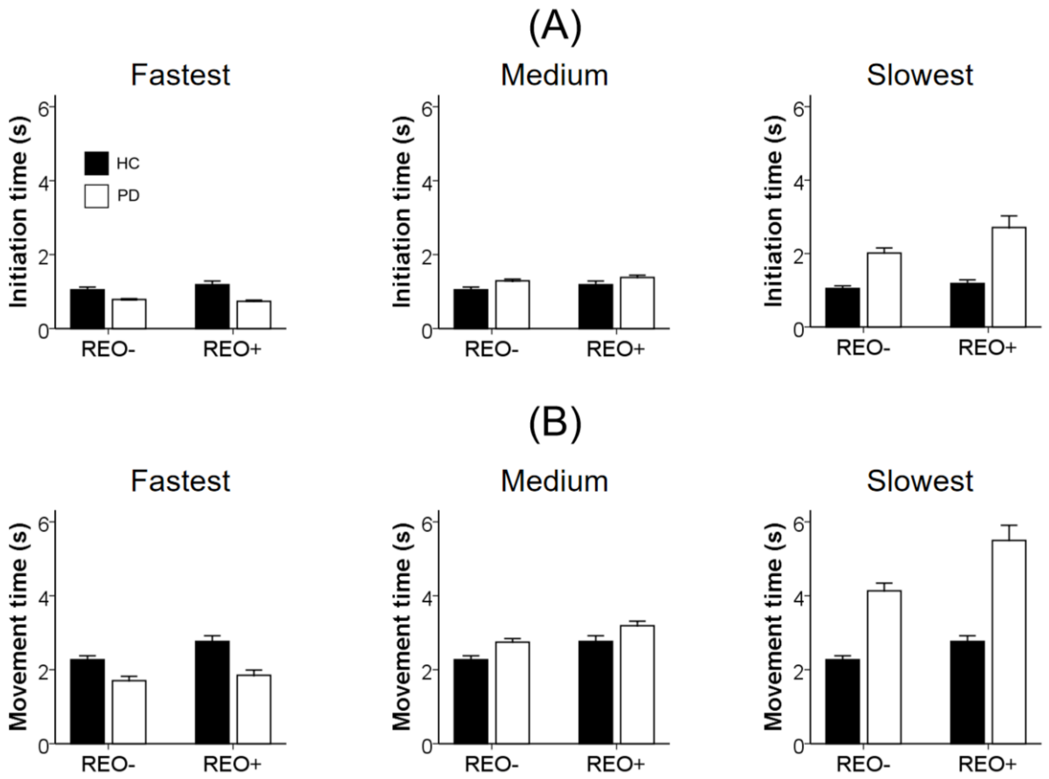
(A)
Fastest
Medium
Slowest
Initiation time (s)
HC
PD
REO-
REO+
(B)
Fastest
Medium
Slowest
Movement time (s)
REO-
REO+
